# Thermal Taster Subgroups and Orosensory Responsiveness Dataset

**DOI:** 10.1016/j.dib.2020.106325

**Published:** 2020-09-20

**Authors:** Margaret Thibodeau, Martha Bajec, Anthony Saliba, Gary Pickering

**Affiliations:** a Department of Biological Sciences, Brock University, St. Catharines, Ontario L2S 3A1, Canada; b Cool Climate Oenology and Viticulture Institute, Brock University, St. Catharines, Ontario L2S 3A1, Canada; c Department of Psychology, Brock University, St. Catharines, Ontario L2S 3A1, Canada; d Charles Sturt University, Wagga Wagga, NSW 2678, Australia; e Graham Centre for Agricultural Innovation, Locked Bag 588, Wagga Wagga, NSW 2678, Australia; f University of the Sunshine Coast, Sippy Downs, Queensland 4556, Australia

**Keywords:** Thermal taste, Thermal tasting, Taste intensity, Orosensory responsiveness, Metallic, Individual differences, Sensory methodology

## Abstract

Thermal taste is a phenomenon whereby some individuals, known as thermal tasters (TT) experience taste sensations when their tongue is warmed or cooled. It was first reported in 2000 by Cruz and Green [1] and since then, most research has focused on comparing TT to thermal non-tasters (TnT; individuals who do not experience thermally-elicited sensations). As TT rate the intensity of taste stimuli higher than TnT, understanding the nature of this difference may help inform how individual differences in taste perception impact consumer liking and consumption of food and beverages. However, as the mechanism(s) underlying thermal tasting are yet to be fully elucidated, it is unclear if TT should be considered a homogeneous group or if subgroups exist. We created a dataset to help determine if the orosensory advantage is universal across all TT, or if it is mainly attributable to one or more subgroups of TT. To this end, the thermal taste screening data of 297 TT from 12 previous recruitment drives (‘cohorts’) was combined. This created the largest dataset of TT reported to date in a single study, allowing for an in-depth analysis of the differences between TT. After training on appropriate scale use, participants were familiarized with common taste and chemesthetic stimuli (sweet, sour, salty, bitter, umami, astringent and metallic). Using a sip-and-spit protocol, participants rinsed with the stimuli and rated the maximum intensity each stimulus elicited on a generalized Visual Analogue Scale (gVAS) or a generalized Labeled Magnitude Scale (gLMS). To account for minor methodological differences between the cohorts, ratings from each cohort were first converted to z-scores before being combined into the overall dataset. Next, participants underwent a series of 12 trials that assessed response to a thermal elicitation device during which each combination of two temperature regimes (warming and cooling) and three lingual sites (tongue tip, 1 cm to left, 1 cm to the right) were examined in duplicate. Participants were asked to rate the maximum intensity of any sensations experienced during each trial. TT were classified into subgroups based on the type of thermally-elicited taste reported (typically sweet, sour, salty, bitter, metallic), the temperature regime during which the sensation was elicited (warming or cooling) and the location on the tongue tested at which the sensation was experienced. Figures are provided that show the mean intensity ratings of aqueous solutions of chemical stimuli and corresponding standard errors for each of the TT subgroups. In addition, the TT Subgroup Naming Conventions provided should allow for a consistent and clear use of terminology across future thermal taste research. Readers are referred to *Homogeneity of thermal tasters and implications for mechanisms and classification*[2] for a full discussion of how these findings inform our understanding of the mechanism(s) underlying thermal taste and the practical implications of methodological differences in determining thermal taste status.

## Specifications Table

**Table:** 

Subject	Sensory Systems
Specific subject area	Individual differences in taste, chemesthetic and thermal perception in the oral cavity.
Type of data	Table Figure
How data were acquired	Thermally-elicited responses were obtained using a thermal elicitation device (TED). The TED is a computer-controlled 64 mm^2^ Peltier device with thermocouple feedback attached to a water-circulated heat sink (Brock University, Machine Shop). See Thibodeau et al [2] for full details.
Data format	Thermal taste classification – Raw: Data is provided as a binary (yes/no), indicating if each participant belongs to specific subgroups. Orosensory Stimuli Intensity – Z-scores: Data converted to z-scores by cohort and combined is available in the attached data set. Figures are also provided showing mean scores by TT subgroup.
Parameters for data collection	A large dataset of TTS screening data was obtained by combining the results of 12 recruitment drives (‘cohorts’). All cohorts were composed of convenience samples recruited from the Brock University student population and surrounding community. As the goal of this data set is to investigate differences within thermal tasters (TT), only data for participants that could be classified as TT was retained for analysis.
Description of data collection	Thermal taste status data was acquired by applying the TED to the edge of participants’ tongues and asking them to rate any sensations elicited on generalized Labelled Magnitude Scales. In a separate task, participants also provided intensity ratings to aqueous solutions of orosensory stimuli (sweet, sour, salty, bitter and umami, astringency, metallic) by rinsing with the sample using a sip-and-spit protocol.
Data source location	Institution: Brock University City/Town/Region: St. Catharines, Ontario Country: Canada
Data accessibility	With the article
Related research article	M. Thibodeau, M.Bajec, A. Saliba & G. Pickering, Homogeneity of thermal tasters and implications for mechanisms and classification, Physiology & Behavior. (2020) 227: 113160.

## Value of the Data

• Thermal tasters (TT) are individuals that experience taste sensations when their tongue is heated or cooled while thermal non-tasters (TnT) do not [3], [4], [5], [6]. Patterns in the types of TT responses based on the temperature regime, location or the type of orosensation provide insights into potential TT subgroups. Identifying these subgroups provide insights into the mechanisms underlying thermal taste. The attached dataset includes extensive TT subtype classification details for a large sample of TT (n = 299).
• These data will benefit researchers interested in understanding variation in human orosensation. TT rate the intensity of taste stimuli higher than TnT [3], [4], [5], [6]. However, it is unknown if the orosensory advantage of TT is universal or if it is driven by a subset of TT. For example, it has been hypothesized that sweet TT rate sweet stimuli (e.g. sucrose) higher than other TT [7,8]. Figures are provided comparing the orosensory responsiveness of several TT subgroups identified by Thibodeau et al [2], to investigate this hypothesis.
• Researchers interested in the thermal taste status phenomenon will have access to a dataset consisting of a large sample of TTs and their responses both to thermal taste elicitation and to aqueous orosensory stimuli. Together, these results can inform future TTS screening protocols, recruitment targets and classification methods.
• TRPM5, a heat-activated cation channel expressed in taste receptor cells, may be involved in the thermally-elicited sweetness experienced by some thermal tasters [9,10]. However, only 30% of participants report thermally elicited sweetness suggesting that additional mechanism(s) may underlie the phenomenon. By providing the raw data, researchers may gain insights into additional genes and/or pathways worthy of study.
• TT rate the intensity of alcoholic beverages and the emotions they elicit higher than TnT [11], [12], [13]. Improving our understanding of how TT should be categorised in future studies (e.g. as a homogeneous group or as discrete subgroups) allows for further insights into the basis of individual differences in food/beverage preferences, intake and diet-related health outcomes.

## 1 Data Description

The primary aim of this manuscript is to visually display the relationship between orosensory responsiveness and TT subgroups. Each figure shows the mean orosensory responsiveness and corresponding standard error to aqueous solutions of sweet, salty, sour, bitter, umami, metallic & astringent stimuli of a TT subgroup compared to TT who are not a part of the subgroup. Readers are referred to the “TT Subgroup Naming Conventions” for full details of the inclusion criteria for each subgroup.

The raw data associated with each of the figures can be found in the accompanying file “Thermal Taster Subgroups and Orosensory Responsiveness Dataset.xlxs”. The dataset includes information on each participants cohort (Column A), a unique identifier for each participant (ID, Column B) and z-scores of mean orosensory responsiveness for six common orosensations (Columns C-I). The remaining columns provided the TT subgroup status of each participant using a binary (Yes/No) system of coding. “Yes” is used to indicate that a participant is part of the TT subgroup listed in the heading, while “No” indicates that they are not. For example, the participant with ID code 14 is a both a Sweet TT and a Sour TT but is not a Salty TT, a Bitter TT, an Umami TT or a Metallic TT. Readers should consult the “TT Subgroup Naming Conventions” for full details of the inclusion criteria of each TT subgroup.

**Fig. 1:**
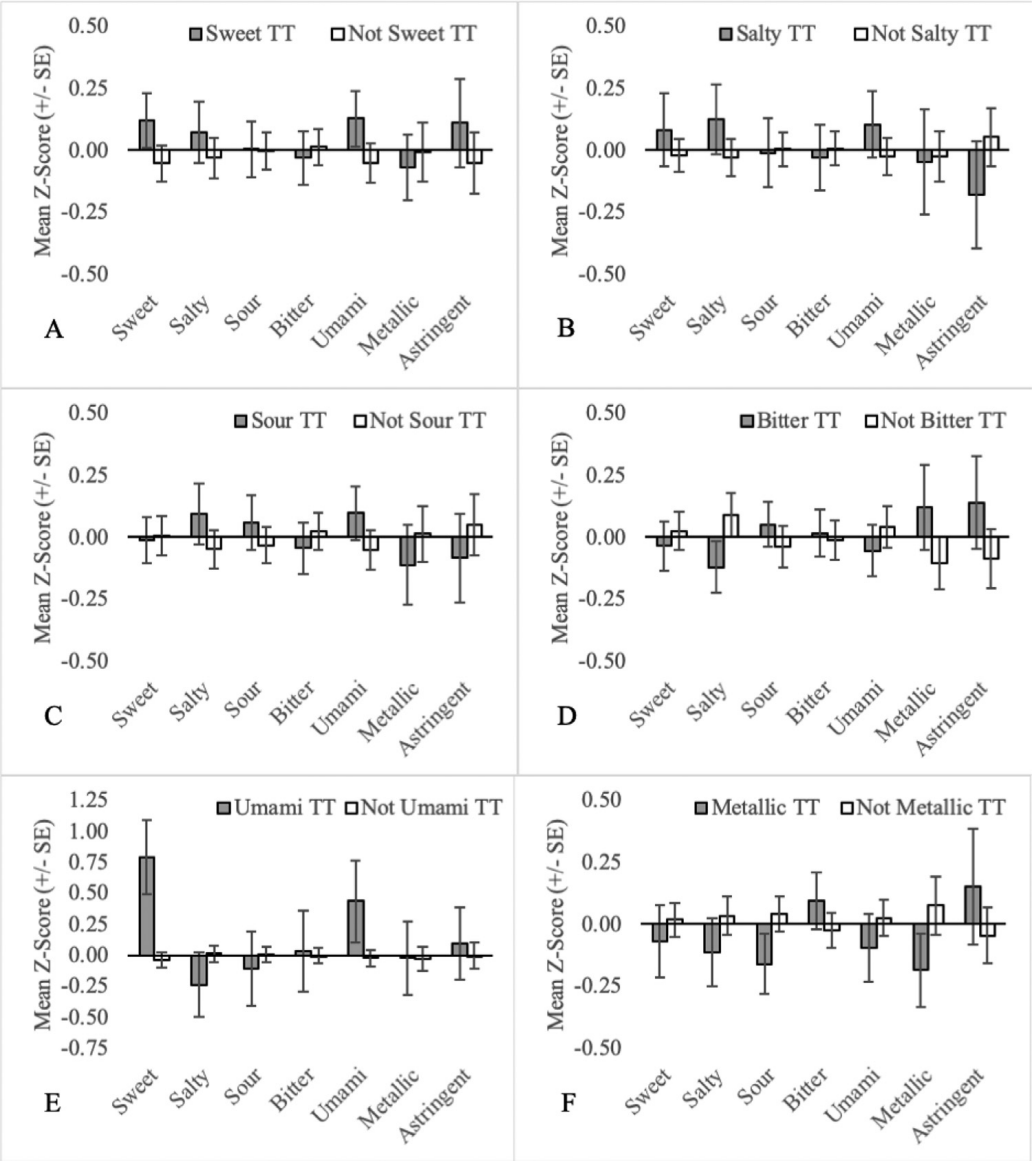
Mean orosensory responsiveness (+/- SE) of sweet TT (A), salty TT (B), sour TT (C), bitter TT (D), umami TT (E) and metallic TT (F) to aqueous solutions (sweet, salty, sour, bitter, umami, metallic & astringent).

**Fig. 2:**
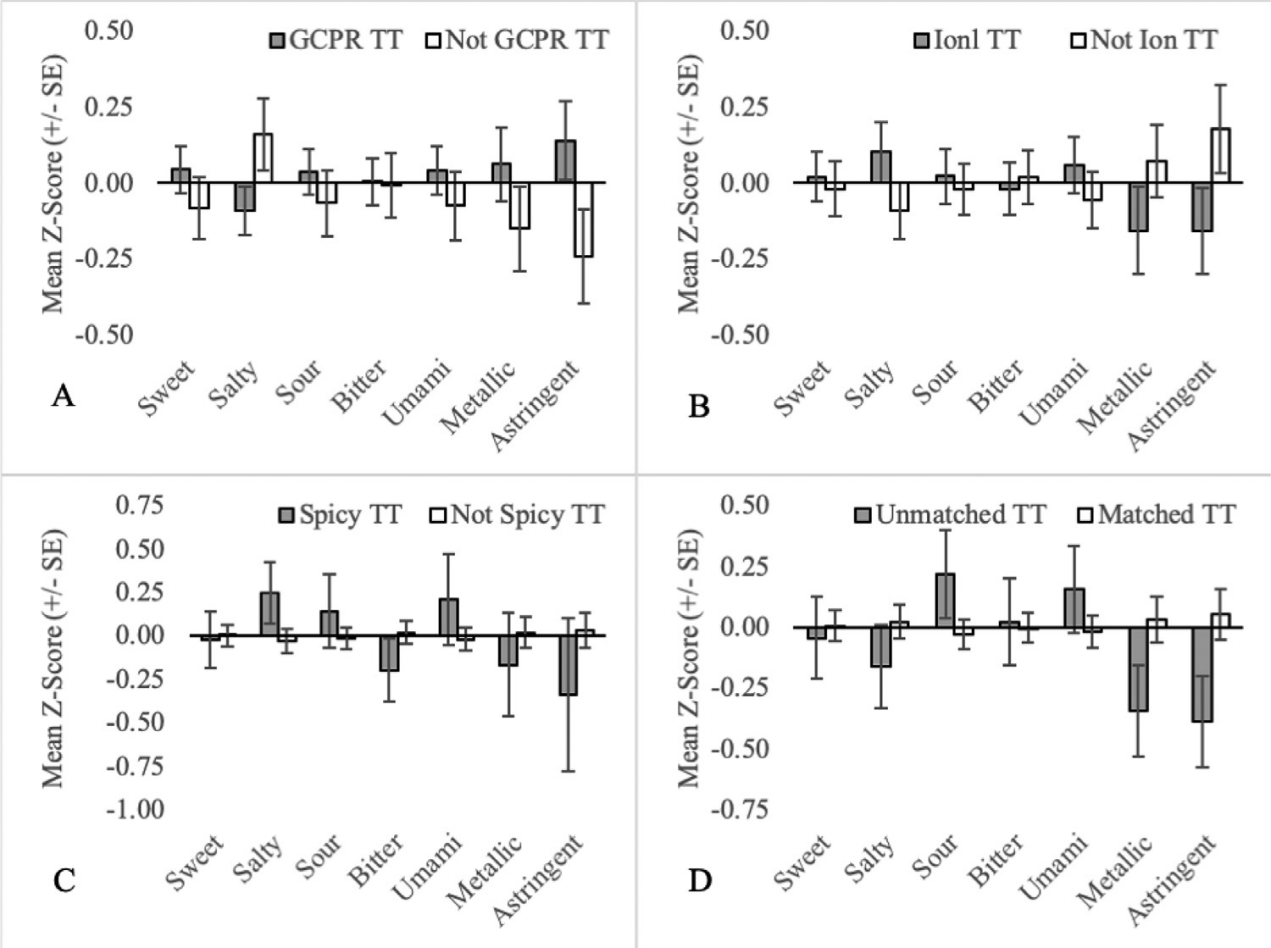
Mean orosensory responsiveness (+/- SE) of GCPR TT (A), Ion TT (B), Spicy TT (C) and Unmatched TT (D) to aqueous solutions (sweet, salty, sour, bitter, umami, metallic & astringent).

**Fig. 3:**
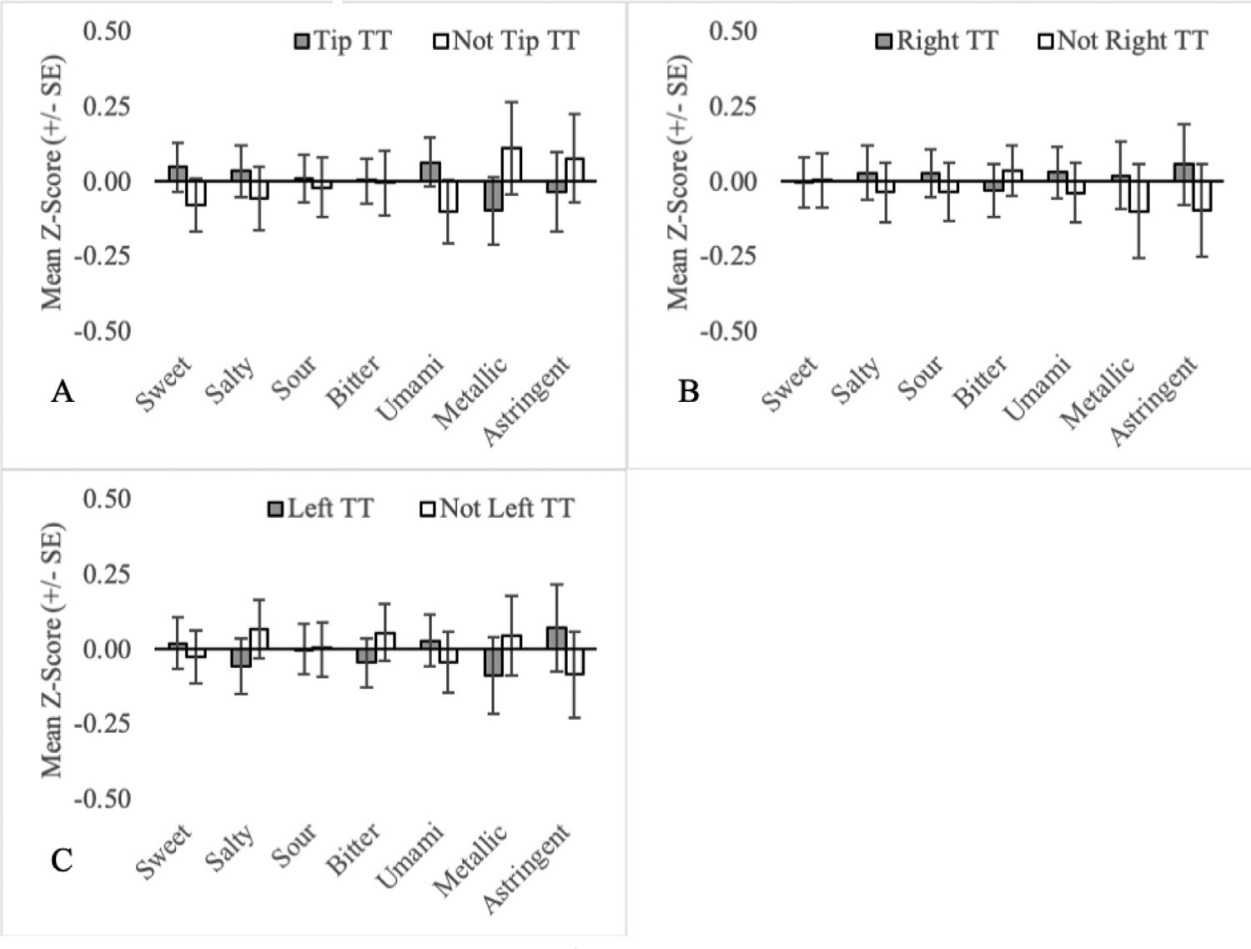
Mean orosensory responsiveness (+/- SE) of tip TT (A), right TT (B) and left TT (C) to aqueous solutions (sweet, salty, sour, bitter, umami, metallic & astringent).

**Fig. 4:**
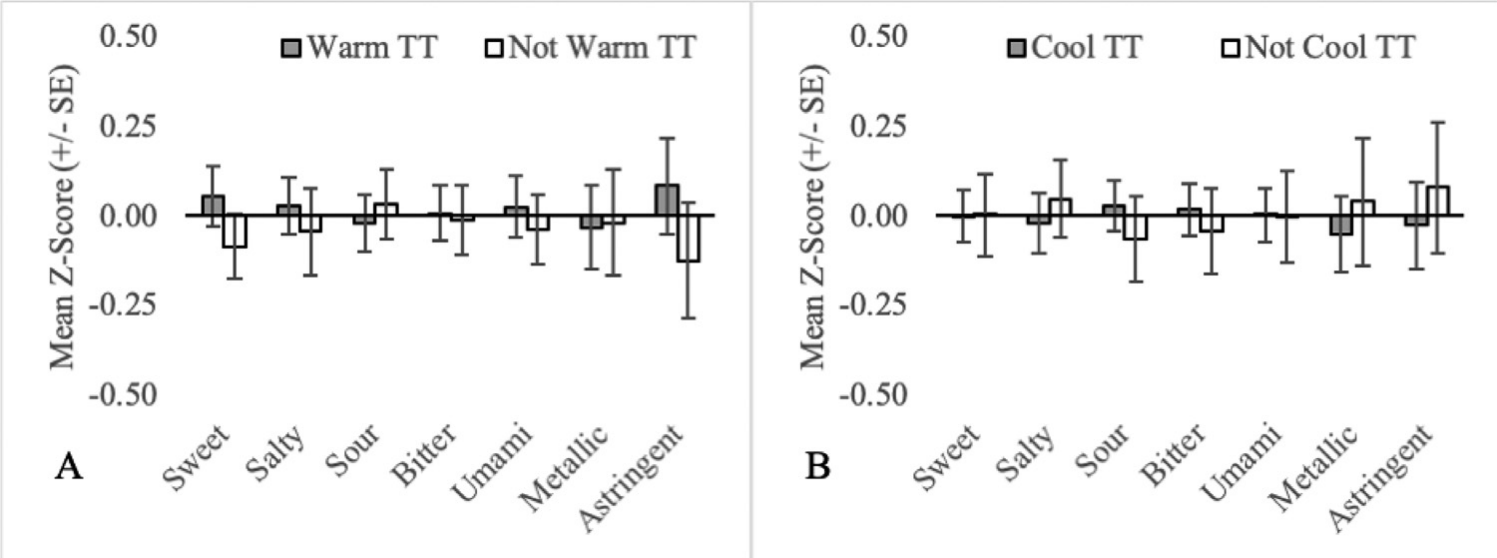
Mean orosensory responsiveness (+/- SE) of warm TT (A) and cool TT (B) to aqueous solutions (sweet, salty, sour, bitter, umami, metallic & astringent).

**Fig. 5:**
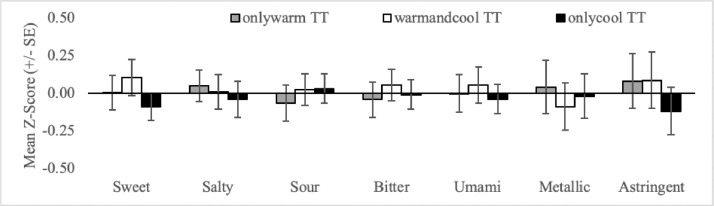
Mean orosensory responsiveness (+/- SE) of onlywarm TT, warmandcool TT and onlycool TT to aqueous solutions (sweet, salty, sour, bitter, umami, metallic & astringent).
(2) Two-factor (n = 254): Each TT belongs to a minimum of three single-factor TT subgroup, suggesting that subgroups based on two factors (e.g. the location and temperature regime) may also be of interest. To this end, Thibodeau et al [2] tested 54 combinations (‘pairs’) of two TT subgroups to determine if a participant that belongs to TT subgroup A was significantly more or less likely to also belong TT subgroup B. Eleven pairs of TT subgroups were positively associated (e.g. Sweet TT were 9 times more likely to also be warm TT) and four pairs were negatively associated (e.g. Sweet TT were 2.2 times less likely to also be Sour TT). For pairs that were positively associated, participants that were members of both subgroups simultaneously (e.g. sweet&warm TT: the participant experienced thermally-elicited sweetness during warming) were compared to the remaining TT (Fig. 6, Fig. 7, Fig. 8; Columns J-T). For pairs that were negatively associated, participants that were not members of either subgroup in the pairs (eg. not sweet TT and/or sour TT) were compared to TT who were members of one or both of the subgroups for the pair (see Fig. 9; Columns U-X).

The data is divided across five tabs as follows:

(1) One-factor (n = 254): Patterns in the types of TT responses to thermal elicitation may provide insights into potential TT subgroups. This tab includes the data for TT subgroups based on the orosensation experienced when the tongue was heated or cooled (see Fig. 1,Fig. 2A, Fig. 2B; Columns O-V),the location at which orosensations were experienced (see Fig. 3; Columns J-L), and based on the temperature regime during which temperature regimes were experienced (see Fig. 4, Fig. 5; Columns M–N).

**Fig. 6:**
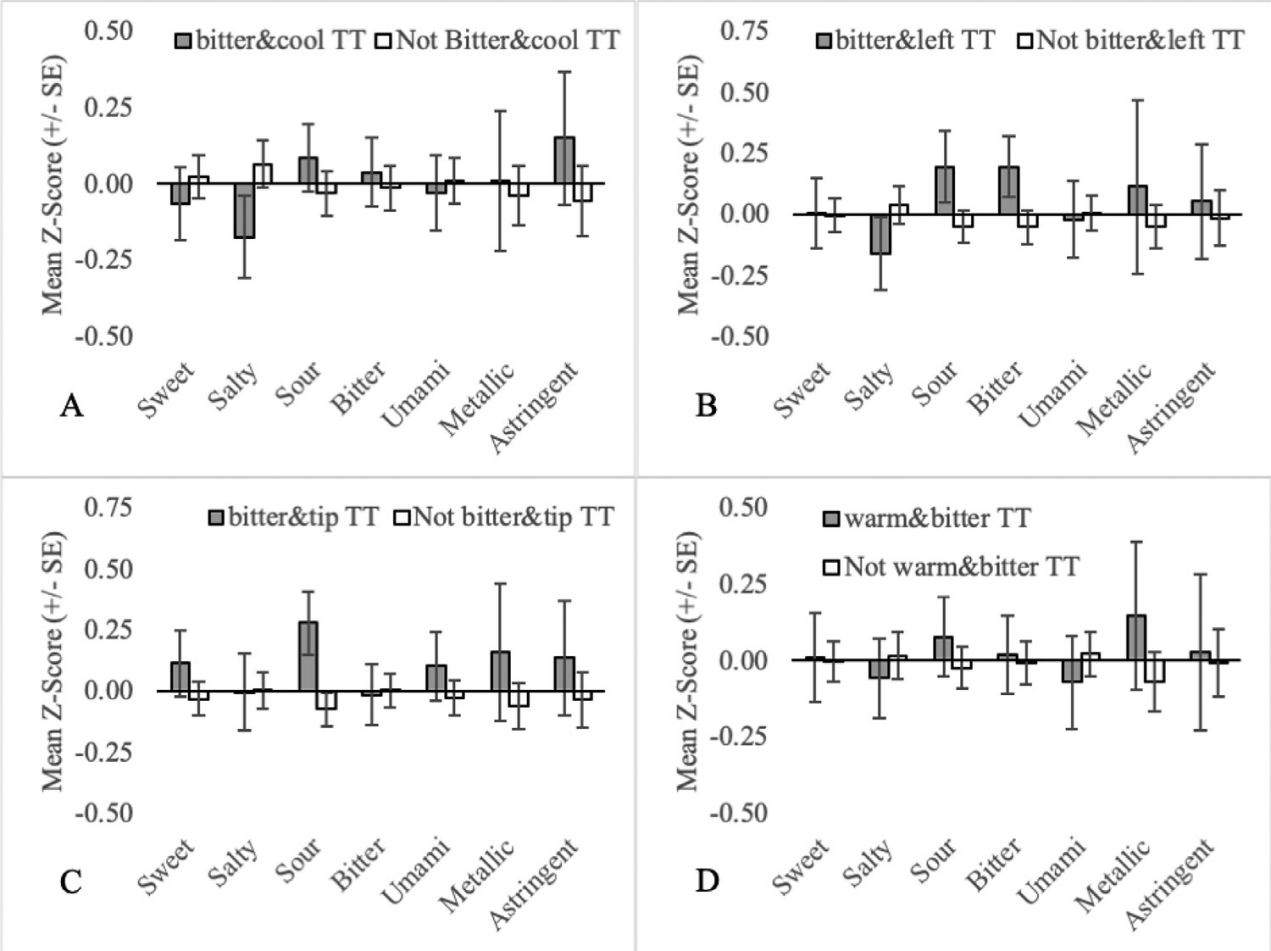
Mean orosensory responsiveness (+/- SE) of bitter&cool TT (A), bitter&left TT (B), bitter&tip TT (C) and warm&bitter TT (D) to aqueous solutions (sweet, salty, sour, bitter, umami, metallic & astringent).

**Fig. 7:**
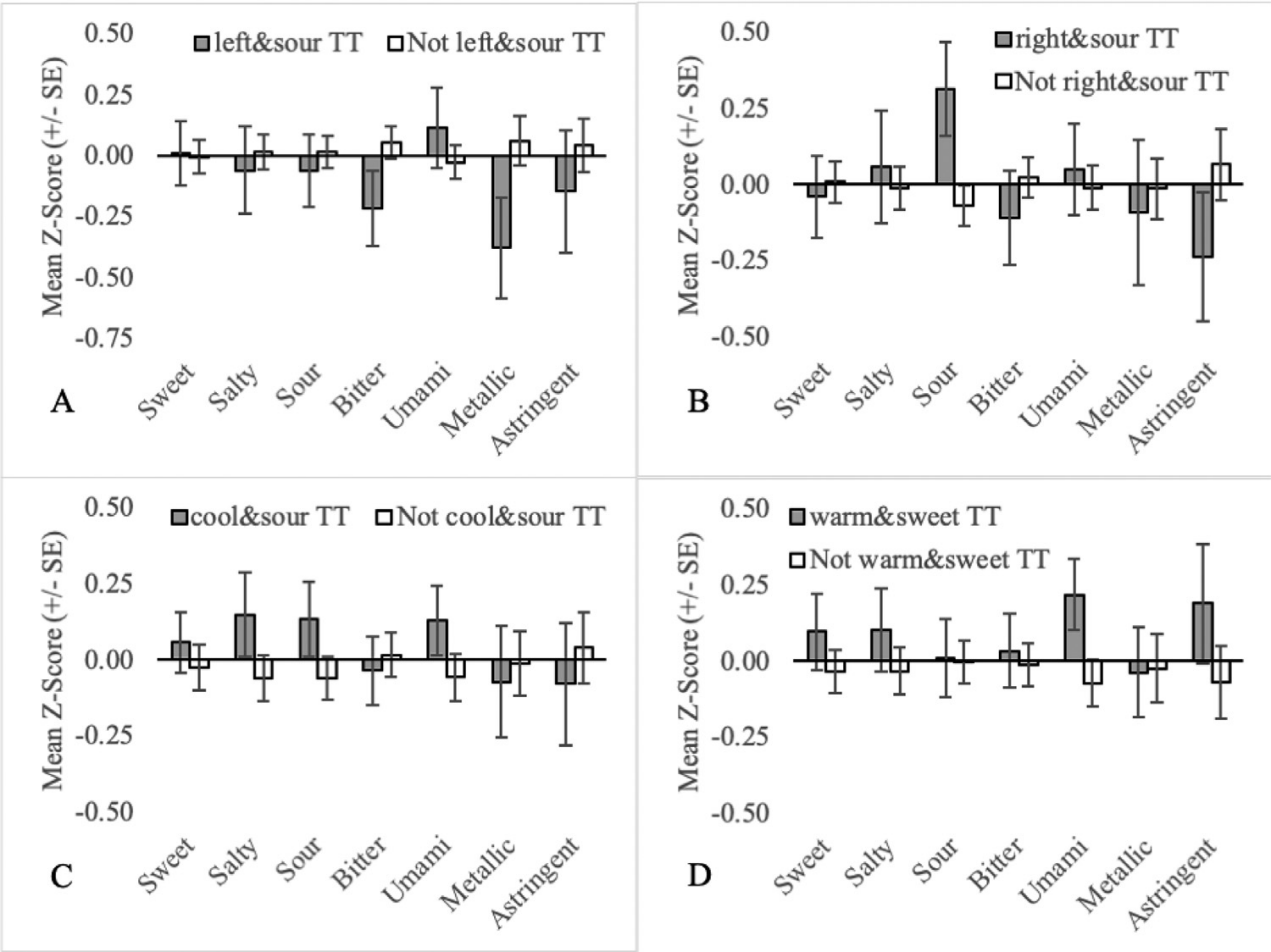
Mean orosensory responsiveness (+/- SE) of left&sour TT (A), right&sour TT (B), cool&sour TT (C) and warm&sweet TT (D) to aqueous solutions (sweet, salty, sour, bitter, umami, metallic & astringent).

**Fig. 8:**
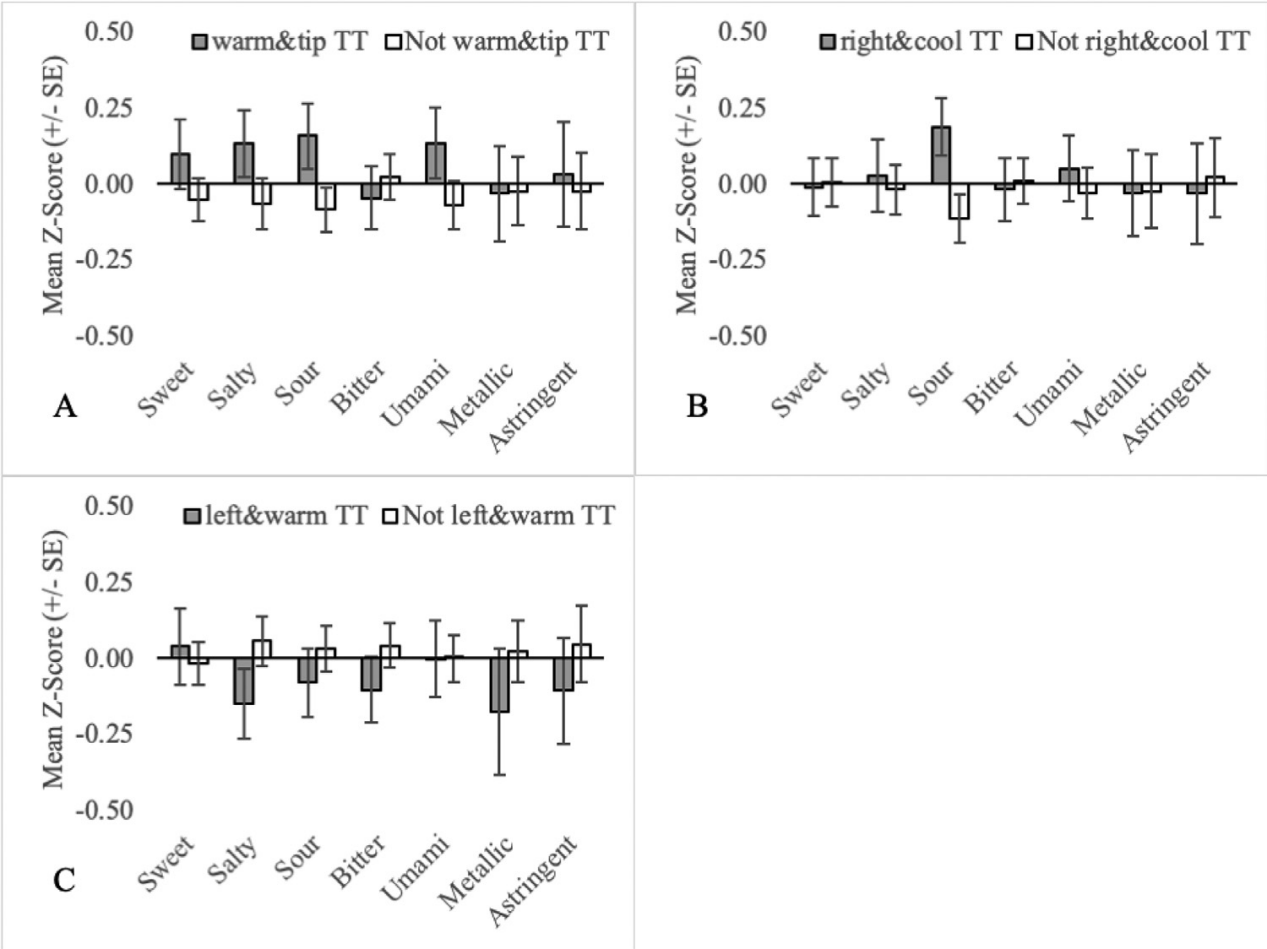
Mean orosensory responsiveness (+/- SE) of warm&tip TT (A), right&cool TT (B) and left&warm TT (C) to aqueous solutions (sweet, salty, sour, bitter, umami, metallic & astringent).

**Fig. 9:**
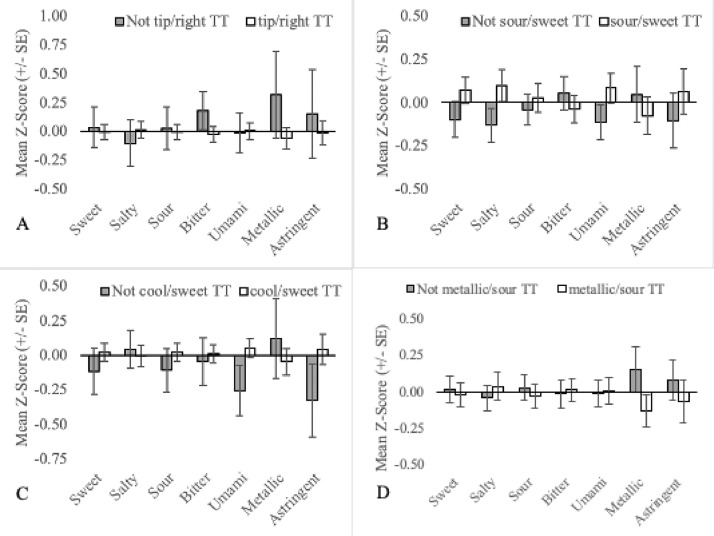
Mean orosensory responsiveness (+/- SE) of not tip/right TT (A), not sour/sweet TT (B), not cool/sweet TT (C) and not metallic/sour TT (D) to aqueous solutions (sweet, salty, sour, bitter, umami, metallic & astringent).
(3) Spicy (n = 265): Includes an expanded dataset with 25 participants who can be classified as Spicy TT if it is included in the list of valid thermally-elicited sensations (Fig. 2C; Column J). Eleven participants IDs (255–265) would not have met the criteria for classification as TT if Spicy was not considered valid (Column K).
(4) Unmatched (n = 286): Includes an expanded dataset with 32 participants (IDs 266–297) who can be classified as Unmatched TT if participants are not required to report the same thermally-elicited sensations in corresponding trials (see Fig. 2D; Column J).
(5) Sample Sizes: As this study is retrospective in nature, not all cohorts were exposed to each of the orosensory stimuli. For convenience, this tab summarizes the sample sizes for each of the TT subgroups.

Please note: As z-scores are calculated using sample means and standard deviations, small differences in the z-scores of some participants exist (1–254) as the number of participants included in the calculations varied. Each participant is assigned the same unique identifier if they are included in multiple tabs of the spreadsheet, for comparison purposes.

## 2 Experimental Design, Materials and Methods

The primary aim of this manuscript was to create a large data set of TT responses. To this end, data from the TTS screening procedures of 12 recruitments drives (‘cohorts’) was combined. 975 participants were recruited from Brock University and the surrounding community, of which 905 completed the study in full. Failure to appropriately use the scales during training led to the exclusion of an additional 124 participants. The final data set includes only the responses for the 297 participants who could be classified as thermal tasters. A description of the experimental design, materials and method is provided next and readers are referred to Thibodeau et al [2] for a comprehensive description.

### 2.1 Data collection

The thermal taste status of all participants was determined based on the protocol of Bajec and Pickering [3], with minor difference in the methods used across the cohorts. These differences reflect changes in best practices, as informed by the developing sensory and thermal tasting literature and differences in study aims across cohorts. The following section briefly describes the methods used to screen for TTS.

After providing informed consent and basic demographic information, participants were training on the appropriate use of two intensity scales, the generalized Visual Analogue Scale (gVAS) and the generalized Labeled Magnitude Scale (gLMS). After, a verbal description of the scale from the researcher, participants were asked to rate the maximum intensity of a series of remembered sensations on each of the scales [3]. Two procedures were implemented to screen for appropriate scale use by the participants. For Cohort 12, the most recent cohort, participants were required to rate the “the brightness of the sun when staring directly at it” more intensely than “the brightness of a dimly lit room”. Cohorts 1–5, 7–11 were required to rate “the pain of biting your tongue” more intensely than the “touch sensation of a pill on your tongue”. As participants from Cohort 6, our first cohort, did not rate the “touch sensation of a pill on your tongue” or “the brightness of a dimly lit room”, no screening for scale use of these participants was performed.

Using a sip-and-spit protocol, participants rinsed with aqueous solutions eliciting common orosensations primarily to aid with the later identification of thermally-elicited sensations (see Table 2 from [2] for full details). All cohorts were presented with exemplars of sweet, sour and bitter. Additional oral sensations included in training were salty (Cohorts 4–12), umami (Cohorts 1–5 & 7–12), metallic (Cohorts 5–7, 10–12) and astringent (Cohorts 1–3,6 & 11). Readers are referred to the data file for a full summary of the sample sizes. All solutions were presented in a randomized order and at room temperature. For each stimulus, participants were presented with 20 ml of each solution in medicine cups or clear wine glasses and asked to swish each solution on their palate for five seconds before expectorating. Participants waited a further 10 s before rating the maximum intensity of the elicited sensation on a gLMS (Cohorts 6 & 12) or gVAS (Cohorts 1–5,7–11) [3]. Each solution was tasted in the presented sequence and participants rinsed with filtered water (Brita, ON, Canada) prior to and after each solution. In order to minimize possible carry-over effects of the metallic and astringent stimuli, unsalted soda crackers (Cohorts 5,7,10–12) or a 5 g/L pectin solution (Cohorts 1–3,6) were provided as palate cleansers. Direct comparison of orosensory responsiveness scores was not possible due to differences in scale, tastants, stimulus concentrations, and/or the number of exposures across cohorts. For all tastants, mean responsiveness scores were calculated for each participant from all replicates. Next, the mean scores from each cohort were converted to z-scores separately. Lastly, the z-scores for each cohort were combined for final analysis.

Thermal stimulation was performed using a 64 mm^2^ computer-controlled Peltier device with a thermocouple feedback attached to a toothbrush-sized water-circulated heat sink (thermode). Two different cycles were used: a warming cycle and a cooling cycle. Warming cycles started at 35°C, then cooled to 15°C before final re-warming to 40°C and holding for 1 s. Participants were only asked to rate the maximum intensity of sensations during the warming phase of the cycle. Cooling cycles started at 35°C, with subsequent cooling to 5°C and holding for 10 s. Participants were asked to report any sensations regardless of when they occurred during the cooling cycle.

Three locations on the edge of the tongue were tested for each participant: the very tip of tongue along the midline, 1 cm to the left from the midline and 1 cm to the right from the midline. A total of 12 runs were performed for each participant in two blocks. Each block consisted of three warming cycles (one per location) followed by three cooling cycles (one per location). A minimum 3 min break was taken between blocks. All participants rated any sensations (heat, cold, sweet, salty, sour, bitter, and other) elicited using a paper ballot with individual gLMS scales for each. For the most recent cohorts (Cohort 5,7,10–12), the paper ballot was modified by adding gLMS scales for umami and metallic.

TTS classification was determined using the methods of Bajec et al [3]. TT were defined as participants who reported the same, valid thermally-elicited taste sensation above weak on the gLMS (> 6 mm) during both replicates of the same location during the same temperature regime (n = 254). Valid thermally-elicited tastes were sweet, salty, sour, bitter, umami and metallic. All other participants (thermal non-tasters and non-classifiable), were excluded from the dataset.

### 2.2 TT Subgroup Naming Conventions

TT were divided into subgroups based on the orosensation(s) reported, the temperature regime(s) and the location of the thermally-elicited orosensation(s). The following conventions were followed in classifying and naming the groups:

(1) Participants who experienced sweetness above ‘weak’ on the gLMS during both replicates for at least one temperature regime and location combination are referred to as sweet TT. Similarly, participants that report a different orosensation are defined as salty TT, sour TT, bitter TT, umami TT or metallic TT based on the orosensation reported.
(2) Participants who reported the same orosensations (sweet, salty, sour, bitter, umami or metallic) above ‘weak’ during both warming replicates for at least one location are referred to as warm TT. Similarly, participants who experience thermally-induced orosensations during cooling are cool TT.
(3) Participants who experienced the same orosensations (sweet, salty, sour, bitter, umami or metallic) above ‘weak’ during both warming and/or cooling replicates at the tongue tip are tip TT. Similarly, participants who experience thermally-induced orosensations on the left or right side of the tongue are defined as left TT or right TT.
(4) At minimum, each participant belongs to three subgroups; one from each of (1–(3) above. However, TT may belong to more subgroups if they experience multiple thermally-elicited orosensations above ‘weak’ or if the sensation(s) is experience at more than one location or during both temperature regimes. Membership of more than one group is designated by an “&” or “/“. The use of each symbol is demonstrated below using the example of a participant who is both a sweet TT and warm TT.
  (a) The “&” symbol indicates that the participant is a member of both groups simultaneously. Thus, a sweet&warm TT reports thermally-induced sweetness during both warming replicates at a minimum of one location.
  (b) A sweet/warm TT could be either a sweet&warm TT or a TT that does not report thermally-induced sweetness during both warming replicates at a minimum of one location. E.g. they could report saltiness during warming and sweetness during cooling.
  (c) Please note: It is not possible to be a warm&cold TT as the warming and cooling regimes were tested during separate trials. Similarly, it it not possible to be a left&right TT, left&tip TT or right&tip TT. However, as multiple orosensations can be elicited in a single trial, it is possible to use the “&” symbol for two thermally-elicited orosensations. For example, a TT that experiences both thermally-elicited sweetness and sourness at the same location and during the same temperature regime is a sweet&sour TT.

(5) TT subgroups defined in (1)–(3) are referred to as single-factor subgroups because only one criterion was used to classify participants. TT subgroups from (4) are referred to as two-factor subgroups.

### 2.3 Figure Generation

The mean orosensory responsiveness z-score (sweet, salty, sour, bitter, umami, metallic and astringent) was plotted for each TT subgroup comparing it to all other TT who were not part of that subgroup (e.g. sweet TT vs not sweet TT). Subgroups examined include single-factor subgroups based on the naming conventions (1; see Fig. 1), (2; see Fig. 4) and (3; see Fig. 3).

Additional TT subgroups were all developed and plotted based on the following criteria:

• Prototypical tastes are broadly divided into two classes based on mechanism. G-protein-coupled receptors (GCPR) are responsible for the perception of sweetness, bitterness and umami while ion channels are responsible for the perception of saltiness and sourness [14]. To assess the importance of mechanism, sweet TT, bitter TT and umami TT are collapsed into a single group called GCPR TT (Fig. 2A). Similarly, salty TT and sour TT were defined as Ion TT (Fig. 2B).
• Three mutually exclusive subgroups based on temperature regime are also recognized. Individuals that experience thermally-elicited tastes during only warming, only cooling or both warming and cooling, are referred to as onlywarm TT, onlycool TT and warmandcool TT, respectively (Fig. 5).
• Two-factor TT subgroups, as outlined in naming convention (4), allow for more precision when defining TT subgroups. This may be important in understanding the association between TTS screening responses and the orosensory advantage of TT. For example, 77 participants are sweet TT but only 64 experience thermally-elicited sweetness during warming. Therefore, by excluding the 13 TT who experience thermally-elicited sweetness during cooling, noise in the dataset may be reduced. As 54 TT subgroups can be established, based on naming convention (4a) alone, a more targeted approach was necessary when selecting two-factor TT subgroups to examine. Two-factor TT subgroups were selected based on the findings of Thibodeau et al [2] who used Fisher's exact tests to test for association between pairs of single-factor TT subgroups. Pairs that occurred together significantly more often than by chance (n = 11) were examined by classifying individuals as factor1&factor2 TT or not factor1&factor2 TT (Fig. 6, Fig. 7, Fig. 8). Pairs that occurred together significantly less often than by chance (n = 4) were used as the basis for TT subgroups by comparing participants that were not factor1/factor2 TT, to those that were (Fig. 9).

While the naming conventions provide a clear definition of a TT, other studies have further expanded their definition of TT. This is possible as the mechanism(s) underlying thermal taste are not well elucidated. The orosensory responsiveness of two additional TT subgroups was investigated by re-classifying participants as follows:

• Consistent with some studies [4,13,15,16], the list of valid thermally-elicited orosensations was expanded to include spicy. Similarly to naming convention (1), participants who reported spicy above ‘weak’ on the gLMS during both replicates for at least one temperature regime and location combination were classified as spicy TT. This was possible as participants were able to rate the intensity of spicy orosensations using the “other” scale provided.
• TT are required to report the same taste sensation across replicate trials in most but not all [4,13,15] studies. The TT dataset was expanded to included Unmatched TT; participants who reported two different thermally-elicited sensations above ‘weak’ on the gLMS during replicate trials and who had previously been classified as thermal non-tasters or non-classifiable.

## Ethics Statement

Informed consent was obtained from all individual participants and all procedures were cleared by the Brock Research and Ethics Board.

## Declaration of Competing Interest

The authors declare that they have no known competing financial interests or personal relationships which have, or could be perceived to have, influenced the work reported in this article.

## Appendix Supplementary materials

Image, application 1
